# Innate Immunity Reimagined: Metabolic Reprogramming as a Gateway to Novel Therapeutics

**DOI:** 10.7150/ijbs.114010

**Published:** 2025-07-28

**Authors:** Chuan-Han Deng, Chen-Tong Wang, Xin Zhou, Xin Chen, Ying Wang

**Affiliations:** 1 Institute of Chinese Medical Sciences and State Key Laboratory of Mechanism and Quality of Chinese Medicine (University of Macau) (MQCM), University of Macau, Avenida da Universidade, Taipa, Macao SAR, China.; 2 Longhua Hospital Shanghai University of Traditional Chinese Medicine, Shanghai, 200032, China.; 3 Department of Pharmaceutical Sciences, Faculty of Health Science, University of Macau, Avenida da Universidade, Taipa, Macao SAR, China.; 4 MoE Frontiers Science Center for Precision Oncology, University of Macau, Avenida da Universidade, Taipa, Macao SAR, China.; 5 Minister Of Education Key Laboratory of Tumor Molecular Biology, Jinan University, Guangzhou 510632, China.

**Keywords:** metabolism programming, cellular respiration, innate immune response, inflammation, mitochondria

## Abstract

The interplay between cellular metabolism and innate immunity critically shapes the body's ability to fight infections, repair tissue, and manage stress. Metabolic reprogramming not only drives innate immune activation but also regulates the resolution of inflammation. Phenotypes of immune cell are closely linked to metabolic shifts that adapt to varying energy demands. However, the precise relationship between perturbations in the cellular respiratory-metabolic axis and the inflammatory response remains a critical field of investigation. In depth understanding of key metabolic pathways, such as glycolysis, NADPH oxidase activity, mitochondrial ROS production, TCA cycle metabolites, and cGAS-STING/AIM2 inflammasome activation, is essential to unravel the complexities of innate immunity. This article highlights the central role of metabolic reprogramming mainly in innate immunity and explores its potential as a therapeutic target for modulating inflammatory response.

## 1. Introduction

Innate immunity serves as the first line of defense against pathogen infection and tissue injury, mobilizing a suite of immune cells, such as dendritic cells, macrophages, and T cells, to engage in a rapid, non-specific response. These cells experience profound transcriptional and translational modifications, with a concurrent metabolic shift to sustain the immediate demands of the immune response. Typically, proinflammatory cells shift from oxidative phosphorylation (OXPHOS) to glycolysis, a metabolic alteration that provides both energy and biosynthetic precursors. The generation of reactive oxygen species (ROS) and mitochondrial signaling pathways are crucial determinants of the inflammatory response and immune cell function. Sustained activation of the innate immune response result in precipitate deleterious conditions, such as cytokine storms or autoimmune diseases 1. Targeting metabolic reprogramming offers a promising strategy for developing novel therapies for inflammatory and autoimmune diseases. This review delineates the intricate steps and pivotal molecules in metabolic programming and sheds light on emerging therapeutic strategies aimed at their regulation.

## 2. Cytoplasmic Metabolic Signaling Hubs

### 2.1. Glycolysis Enzymes

Under normoxic conditions, the Warburg effect induces a switch of metabolism from OXPHOS to glycolysis, favoring aerobic glycolysis for ATP generation. Immune cells resort to Warburg metabolism upon encountering inflammatory stimuli, a strategy that underpins their resistance to lactate-mediated suppression and supports cellular proliferation 2. Dendritic cells, for instance, augment glucose consumption and engage in Warburg metabolism following Toll-like receptor (TLR) activation 3. Glycolysis and the pentose pathway become the predominant source for ATP production in T cells and M1 macrophages, sidelining the tricarboxylic acid (TCA) cycle 4. The accumulation of lactic acid not only aids in restoring metabolic equilibrium but also can induce a phenotypic switch in immune cells towards a quiescent state, which marks the cessation of the immune response 5.

**Figure 1:**
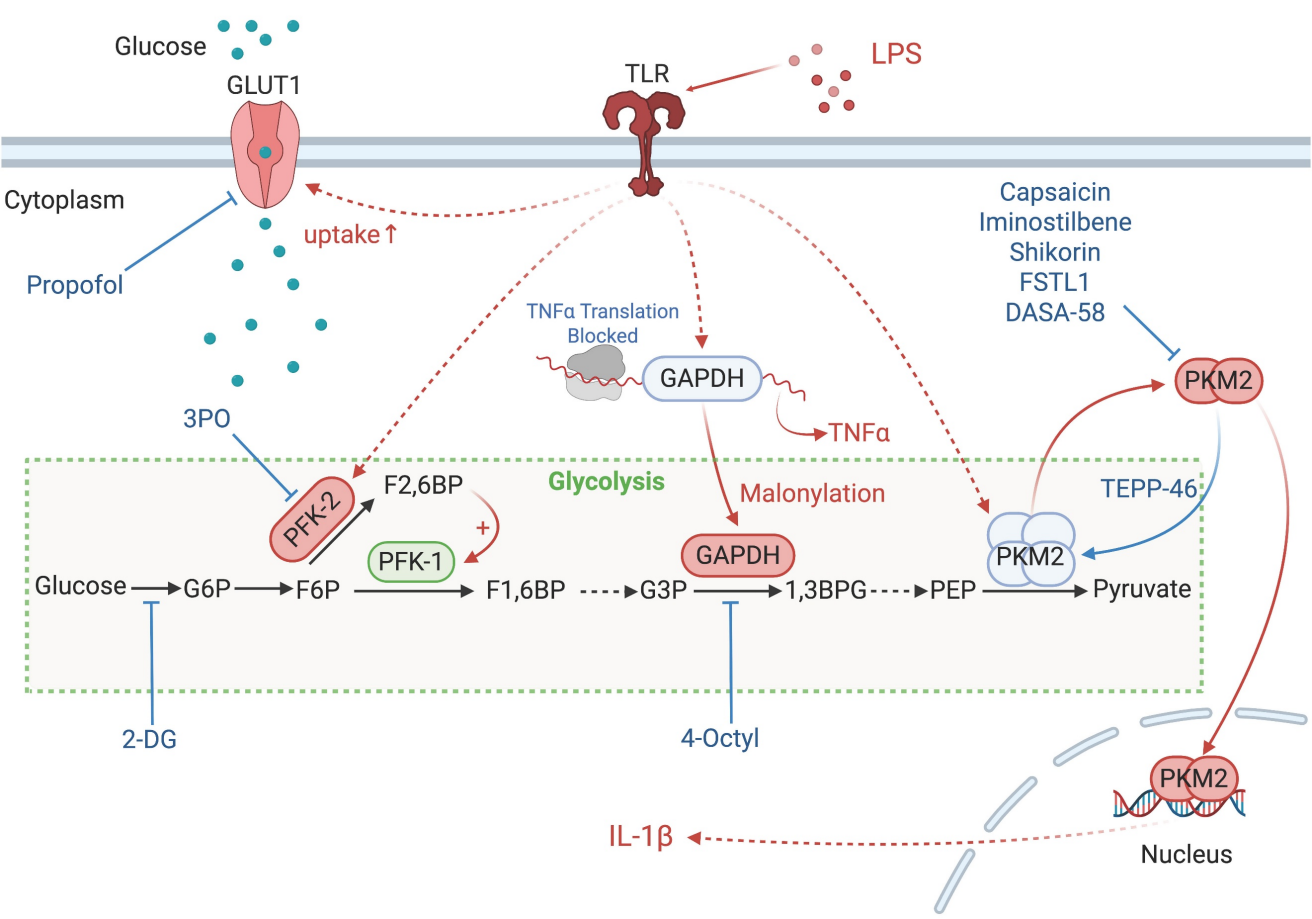
The enzymes involved in glycolysis play important role in innate immune response, either directly or indirectly. Certain compounds have anti-inflammatory effects by inhibiting enzyme activity or altering enzyme conformation. Red arrows indicate pro-inflammatory effects; blue arrows indicate anti-inflammatory effects.

Glycolysis is an intricately controlled sequence of biochemical reactions (Figure 1). The rate of cellular glucose absorption is largely governed by glucose transporters (GLUT). Activation of T cells necessitates a prompt and robust upregulation of GLUT1, a requirement not shared by quiescent peripheral T cells for their survival 6. During *Streptococcus pneumoniae* infection, the AIM2 inflammasome is triggered by GLUT1-mediated glycolysis, thereby intensifying pulmonary fibrosis 7. Sepsis-induced Warburg effect via GLUT1 can lead to the apoptotic demise of CD4^+^ T cells, precipitating a collapse of immune function 8. In models of encephalomyelitis and autoimmune colitis, glucose uptake via GLUT3 modulates glucose oxidation and ATP-citrate lyase-dependent acetyl-CoA synthesis in the mitochondria, influencing the epigenetic reprogramming of inflammatory genes in T helper (T_h_)17 cells 9.

The enzymatic transformation of glucose into glucose-6-phosphate, catalyzed by hexokinase (HK) 1 to 4, marks the onset of aerobic glycolysis. The dissociation of HK1 from mitochondria and its binding to S100A8/A9 promotes iNOS-dependent nitrosylation and GAPDH inactivation. This redirects glycolytic flux to the pentose phosphate pathway and enhances nitric oxide signaling. This metabolic shift leads to oxidative stress and low-grade chronic inflammation that contribute to tissue damage in diabetic neuropathy and aging 10. In LPS-primed macrophages, HK1 detects cytosolic N-acetylglucosamine, a peptidoglycan metabolite which triggers the dissociation of HK1 from the mitochondria. This inhibits enzyme activity of HK1 and leads to elevated ROS, which act as Signal 2. Signal 2 then drives the assembly of the NOD-like receptor family pyrin domain containing 3 (NLRP3) inflammasome, resulting in inflammation 11. HK2 expression alteration is notably significant in activated T cells. The inhibition of HK2 by bacterial peptidoglycan-derived N-acetyl glucosamine results in its detachment from the mitochondrial outer membrane and the subsequent assembly of the NLRP3 inflammasome. Interfering with glycolysis via the addition of glucose-6-phosphate, the enzymatic product of HK2, nullifies its pattern recognition receptor function 11.

Glucokinase, a hexokinase isozyme with lower affinity for glucose, converts glucose to glucose-6-phosphate primarily in hepatocytes and pancreatic beta cells. The initiation of glucokinase-mediated glycolysis leads to its interaction with actin, which promotes cytoskeletal reorganization and the migration of T regulatory cells. This migratory response is driven by glucokinase expression upregulation via the phosphoinositide 3-kinase (PI3K)-mammalian target of rapamycin (mTOR) complex 2 signaling axis 12.

The third step of glycolysis is catalyzed by phosphofructokinase-2 (PFK-2), which facilitates conversion of fructose-6-phosphate to fructose-2,6-bisphosphate (F2,6BP). PFK-1 activity is allosterically upregulated by F2,6BP 13, which is synthesized from fructose-6-phosphate by fructose-6-phosphate-2-kinase (PFK2/PFKFB3). Genetic variants such as rs646564 in the PFKFB3 gene reduce glycolytic ATP production, resulting in impaired generation of ROS outburst. This leads to defective phagocytosis and poor fungal clearance in human macrophages 14.

Glyceraldehyde 3-phosphate dehydrogenase (GAPDH) undertakes the sixth step of glycolysis to catalyze the oxidation of glyceraldehyde-3-phosphate to 1,3-biphosphoglycerate. Malonylation at Lys213 on GAPDH disrupts its interaction with AU-rich mRNA elements, such as TNFα, resulting in the release of these transcripts for translation and the subsequent activation of pro-inflammatory signaling pathways. Concurrently, this modification impairs GAPDH's glycolytic enzymatic activity, and reprograms glycolysis to meet the energy demands of the macrophages during inflammation 15. GAPDH also plays a role in T cell activation and glycolysis, with its direct interaction with the AU-rich element in the 3' untranslated region on interferon (IFN)-γ mRNA 16.

Pyruvate kinase isoezymes M2 (PKM2) orchestrates the final and rate-limiting step of glycolysis by catalyzing the transformation of phosphoenolpyruvate to pyruvate when in its active tetrameric state. In its dimeric configuration, PKM2 translocate to the nucleus to activate transcription factor 2, thereby enhancing LPS-induced pyroptosis in microglia 17. Additionally, when associated with HIF-1α in the nucleus, PKM2 initiates the transcription of interleukin (IL)-1β, inhibits glycolysis and shifts macrophages towards the M2 phenotype under LPS and *Salmonella typhimurium* exposure 18. PKM2-mediated glycolysis also facilitates the phosphorylation of eukaryotic translation initiation factor 2-alpha kinase 2, activating the AIM2 and NLRP3 inflammasomes in macrophages, a critical process in lethal endotoxemia and polymicrobial sepsis 19. Pharmacological intervention that activates PKM2 to its tetrameric state impedes its nuclear translocation and subsequent transcription of pro-inflammatory genes. The allosteric PKM2 activator TEPP-46 mitigates CD4^+^ T cell-driven autoimmune and inflammatory responses in autoimmune encephalomyelitis models 20. In contrast, sulfenylation of PKM2 impedes its tetramerization and reduces its enzymatic activity, which in turn augments glycolytic flux and the accumulation of harmful glucose metabolites 21.

Lactate dehydrogenase A (LDHA) catalyzes the conversion of pyruvate to lactate, a process that succeeds aerobic glycolysis. Elevated LDHA expression favors aerobic glycolysis, sustaining acetyl-coenzyme A concentrations necessary for histone acetylation, which in turn modulates epigenetic control of IFN-γ production in T cells upon activation. A deficiency in LDHA, however, can lead to PI3K-mediated dephosphorylation of Akt, reducing T cell-mediated immunity in mice challenged with bacterium *Listeria monocytogenes* 22.

### 2.2. Nicotinamide Adenine Dinucleotide Phosphate (NADPH) Oxidase

The NADPH oxidase (NOX) family, along with the mitochondrial electron transport chain (ETC), are primary sources of reactive oxygen species (ROS) and directly generate ROS such as superoxide and hydrogen peroxide. To date, the NOX family has been expands to encompass seven isoforms, NOX1 to NOX5, along with dual oxidase (DUOX)1-2, each with unique tissue distribution and physiological functions 23.

The NOX2 complex, along with its regulatory subunits p40^phox^, p47^phox^, and p67^phox^, was initially characterized as the primary component of the phagocyte oxidative burst 24. Upon infection, these regulatory subunits translocate to the membrane, and form the active oxidase complex together with gp91^phox^ and p22^phox^. NOX2 facilitates the generation of superoxide via a biphasic electron transfer process, essential for pathogen eradication 24. This activation promotes a significant upsurge in both OXPHOS and glycolysis 24. A missense mutation in the neutrophil cytosolic factor 2 gene, which encodes p67^phox^, has been linked to early-onset IBD 25. Conversely, a deficiency in NOX2 predisposes individuals to autoimmunity and elevate systemic lupus erythematosus risk 26. On the other hand, hyper activation of NOX2 can lead to oxidative stress, contributing to chronic inflammation and tissue damage. Targeted reversible inhibitors that hinder p47^phox^ and p22^phox^ interactions effectively mitigate NOX2-induced oxidative stress 27.

Compounds like LDC7559 and its more efficacious derivative, NA-11, target the AMP/ADP allosteric site on phosphofructokinase-1 liver type (PFKL). This interaction inhibits glycolysis and the subsequent pentose phosphate pathway, diminishing the NOX2-dependent oxidative burst and the defense capability of neutrophils, thereby curtailing tissue damage 28. Additionally, during *Staphylococcus aureus* infection, NOX2 generates ROS, which alkalinizes phagosomes by consuming protons during the conversion of oxygen to superoxide (O₂⁻). This counteracts the acidifying activity of the V-ATPase proton pump. However, caspase-1 subsequently cleaves subunits of the NOX2 complex, reduces ROS production and allowing V-ATPase-driven acidification to proceed. This enhances the killing of Gram-positive bacteria via lysosomal enzymes 29.

Inhibition of NOX4 bolsters the endothelial cell barrier in sepsis and mitigate acute lung injury 30. GKT137831, a NOX4 inhibitor, is undergoing clinical evaluation for idiopathic pulmonary fibrosis (Clinical trial No. NCT03865927), type 2 diabetes (NCT02010242), and primary biliary cholangitis (NCT03226067). While ablation of NOX4 enhances liver regeneration in mice 31, yet it appears to confer protection against tissue damage due to fibrogensis in chronic intestinal inflammation 32. Given NOX4's multifaceted roles in different disease states, pharmacological targeting requires precise tailoring to minimize off-target effects.

NOX5, an oxidase primarily expressed during monocytes differentiation to dendritic cells and implicated in vascular remodeling and calcification 33. NOX5 expression in podocytes is linked to the heightened ROS and pro-inflammatory cytokine production via activation of IL-1R-associated kinases (IRAK)-1, IRAK-4 34, and protein kinase C-α signaling 35. The broad-spectrum NOX inhibitor APX-115 enhances pancreatic beta-cell functionality and mitigates diabetic nephropathy in NOX5 overexpressing transgenic mice 36.

DUOX1 and DUOX2, initially identified in the thyroid, are crucial for thyroid hormone biosynthesis. In the lungs, IL-1β and ROS, generated by DUOX1, constitute a unified epithelial response to microbial infections 37. DUOX2's primary function is to protect against pathogenic gut microbiota by producing hydrogen peroxide 38. Notably, a monoallelic exonic variant of DUOX2 correlates with very early-onset IBD 39, and mutations in DUOX2 are associated with increased colonic IL-17C levels and risk of IBD 40.

### 2.3. Hypoxia-Inducing Factor-1α (HIF-1α)

HIF-1α transcription is principally regulated by nuclear factor kappa B (NF-κB) pathways in response to hypoxia 41. The stability and subsequent nuclear translocation of HIF-1α are crucial in redirecting cellular metabolism towards glycolysis. During neutrophil-mediated oxidative burst, the glycerol 3-phosphate pathway is essential in preserving mitochondrial integrity and supporting glycolysis, thus facilitating HIF-1α stabilization 42. The use of FG-4592 to stabilize HIF-1α diminishes both glycolytic metabolites and cytokine production in alveolar macrophages during acute lung injury 43. Conversely, HIF-1α genetic ablation reduces glycolysis and curtails pro-inflammatory mediator production in macrophages, which has implications in systemic lupus erythematosus 44. Additionally, Wnt ligand stimulation enhances the interaction between β-catenin and HIF-1α, leading to a surge in HIF-1α levels and a subsequent pro-inflammatory response in macrophages from patients with COVID-19 45.

### 2.4. PI3K-Akt Signaling in Metabolic Regulation and Immune Cell Activation

The activation of naïve T cells is precipitated by the engagement of T cell receptor (TCR) complexes with co-stimulatory signals such as CD28, IL-2, and IL-7. The TCR complex influences the Myc pathway and the PI3K-Akt signaling signaling cascade 46. Myc is indispensable for the metabolic reprogramming of T cells, with its absence impeding the induction of glycolytic flux upon T cell activation. Proteins associated with glycolysis and oxidative metabolism are markedly increased during the initial activation of naïve T cells 47. For example, GLUT1 expression in activated T cells, regulated by PI3K-Akt signaling, corresponds with an adaptive increase in glucose metabolism. The reactivation of memory T cells similarly relies on CD28-mediated PI3K-Akt signaling for GLUT1 expression, which is critical for their metabolic demands 48.

## 3. Mitochondrial Metabolic Signaling Hubs

### 3.1. Mitochondria Generated ROS

Mitochondria act as the principal loci for aerobic respiration and serve as the energy biosynthesis powerhouses within eukaryotic cells. The ETC hosts the OXPHOS process that facilitates ATP synthesis, the predominant energy molecule. Electron transit through the ETC establishes a proton gradient across the inner mitochondrial membrane, which, upon reacting with oxygen, generates ROS within the ETC.

Electrons from nicotinamide adenine dinucleotide (NADH) enter the ETC at mitochondrial complex I. An elevated NADH/NAD^+^ ratio within the mitochondrial matrix allows for the interaction of molecular oxygen with reduced flavin mononucleotide (FMN), resulting in the production of the superoxide anion (O_2_^-^), which is liberated into the mitochondrial matrix. Subsequently, the reduction of ubiquinone and alterations in mitochondrial membrane proton concentration induce a reverse electron transport chain (RET), driving electrons back towards complex I and fostering additional O_2_^-^ generation 49. Complex I impairment impairs NADPH production and enhances the inflammatory response due to ROS accumulation 50.

Complex III is another significant contributor to mitochondrial ROS production. The O_2_^-^ generated by complex III primarily enters the inner mitochondrial membrane space, while the H_2_O_2_ formed post-disproportionation permeates the matrix 51. A deficit in complex III function leads to heightened DNA methylation and suppresses the expression of genes critical for the immunosuppressive function of regulatory T (T_reg_) cells without compromising T_reg_ cell proliferation or viability 52. ROS originating from complex III also precipitate the depletion of NAD^+^ levels and intensify DNA damage, processes crucial` for macrophage activation 53.

When both complexes I and complex III are inhibited, complex II becomes the predominant source of ROS 54. Upon LPS stimulation, macrophages exhibit an increase in mitochondrial succinic acid oxidation and membrane potential due to complex II-mediated elevated mitochondrial ROS production. Targeting succinate dehydrogenase within complex II can mitigate ROS generation, dampen the macrophage inflammatory response, and reduce LPS-induced lethality in mice 55. Itaconate, a metabolite produced by activated macrophages, acts as an inhibitor of succinate oxidation at complex II, modulating macrophage metabolism and attenuating inflammation in models of ischemia-reperfusion injury in Irg1^-/-^ mice 56. Cardiolipin biogenesis impedes the assembly of complex II, triggering lysosome-mediated degradation of this complex following LPS exposure in macrophages 57.

Mitochondrial-derived ROS play a pivotal role in modulating the innate immune response 58. They facilitate the relocation of NLRP3 to the mitochondria-associated ER membrane, where it attracts both apoptosis-associated speck-like protein containing a CARD (ASC) and pro-caspase-1, leading to inflammasome activation. Mitochondrial ROS (mtROS) trigger cleavage and oligomerization of the N-terminal domain of gasdermin D, enabling its insertion into the mitochondrial membrane to form pores. These pores amplify mtROS release, which then activates the RIPK1/RIPK3/MLKL necroptotic pathway and drive necroptotic cell death during *Pseudomonas entomophila* infection 58. Excessive mtROS stemming from dysfunctional mitochondria instigate the assembly of the NLRP3 inflammasome. TLR7/8 agonists, such as imiquimod and CL097, impede the activity of quinone oxidoreductases NQO2 and complex I of the mitochondria, thereby heightening intracellular ROS triggering NLRP3 inflammasome activation independent of K^+^ efflux 59. Conversely, curtailing mitochondrial ATP synthesis and DNA replication can avert NLRP3 inflammasome initiation in alveolar macrophages in acute respiratory distress syndrome induced by LPS or SARS-CoV-2 infection 60. While obstructing the mitochondrial ETC can diminish the NLRP3-driven inflammatory cascade, the mitochondrial metabolite phosphocreatine activates the NLRP3 inflammasome in an ATP-dependent manner, irrespective of ROS generation 61.

### 3.2. Mitochondrial Dynamics in Immune Cell Fate and Inflammation

Mitochondrial dynamics is one of the key determent factors in metabolic programming and T cell destiny. Mitochondrial fusion proteins like OPA1 enhance OXPHOS in memory T cells by promoting fused mitochondrial networks and remodeling cristae structure, which optimizes ETC efficiency. This tight ETC coupling sustains high ATP production, supporting the metabolic demands and longevity of memory T cells 62.

Mitophagy, a specialized autophagic mechanism, selectively eliminates malfunctioning or surplus mitochondria and is instrumental in modulating inflammatory responses. FUNDC1, a receptor essential for mitophagy, ensures mitochondrial quality control under normal conditions, and its disruption worsens diet-triggered obesity and metabolic dysfunction 63. Mitophagy prevents NLRC4 activation during *Pseudomonas aeruginosa* infection by removing mitochondria damaged by the type III secretion system (T3SS), thereby reducing mtROS and oxidized mtDNA release. This blocks the cytosolic accumulation of oxidized mtDNA, which is required for NLRC4 inflammasome oligomerization and activation, excessive ROS generation, mitochondrial DNA (mtDNA) release, and subsequent activation of the NLRC4 inflammasome in macrophages 64. In intestinal macrophages, the deletion of IL-10 or its receptor prolongs mTOR pathway signaling, exacerbating inflammasome activity and intensifying intestinal inflammation 65.

The strategic induction of mitophagy through small-molecule agents presents great potential in regulating inflammatory response. Compounds such as rapamycin and resveratrol mitigate NLRC4 inflammasome activation by facilitating mitophagy, thereby clearing damaged mitochondria in mouse bone marrow-derived macrophages (BMDMs) 64. Similarly, andrographolide, the main active substance first isolated from *Andrographis paniculata*, obstructs the advancement of colitis and associated cancers by inhibiting the NLRP3 inflammasome via mitophagy in mouse models 66.

### 3.3. TCA Cycle Metabolites

Mitochondrial metabolism plays a pivotal role in immune regulation, particularly through the tricarboxylic acid (TCA) cycle. The TCA cycle, or termed as the Krebs cycle, represents a fundamental process in biosynthesis and cellular energy production. In immune cells, intermediates of the TCA cycle serve a dual function: they are vital for ATP generation and act as signaling molecules that influence immune responses. For example, a low α-ketoglutarate/succinate ratio leads to proinflammatory state of macrophages, whereas a high α-ketoglutarate/succinate ratio facilitates the tissue repair phenotype of macrophages 67. The inhibition of succinate oxidation by dimethyl malonate, in turn, drives the proinflammatory phenotype of macrophages 55.

One noteworthy endogenous metabolite derived from the TCA cycle-derived is itaconate, which is generated through the decarboxylation of cis-aconitate. Itaconate suppresses inflammatory responses by inhibiting activity of succinate dehydrogenase 56, 68 and to interact directly with Cys151, 257, 288, 273 and 297 on KEAP1 69. The covalent binding of itaconate and KEAP1 then enables increased expression of nuclear factor erythroid 2-related factor 2 (Nrf2) downstream anti-oxidant and anti-inflammatory genes 69. Moreover, the cell permeable derivative of itaconate, 4-octyl itaconate, offers protection against lethality and systemic inflammation induced by LPS 69. Treatment with glucocorticoids facilitates the interaction of glucocorticoid receptor with pyruvate dehydrogenase complex, and then elevates the TCA cycle-dependent production of itaconate and interfere with the production of proinflammatory cytokines 70. This illustrates how metabolic pathways can directly influence immune cell behavior and responses, emphasizing the complex interconnection between metabolism and immunity.

### 3.4. Mitochondrial DNA (mtDNA) Triggered Inflammatory Response

mtDNA, positioned in proximity to the ETC, acts as a principal source of mitochondrial reactive oxygen species (mtROS). mtROS, such as hydroxyl radicals, oxidize mtDNA, generating strand breaks and 8-oxoguanine adducts that structurally mimic pathogen-derived DNA. When released into the cytoplasm, oxidized mtDNA is recognized by cGAS/STING as a "non-self" danger signal and leads to subsequent activation of innate immune pathways and conferring immunogenicity 71. mtDNA is rich in hypomethylated CpG motifs, identifiable by pattern recognition receptors (PRRs) like cGAS-stimulator of interferon genes (STING), TLR9, and the AIM2 inflammasome 72, 73. Experimental intra-articular injection of mtDNA in mice provokes a pro-inflammatory response 74. Mitochondrial ROS can also impair mtDNA synthesis by diminishing the level of mitochondrial transcription factor A, intensifying the severity of ischemic acute kidney injury 75.

Cells expel defective mitochondria through mitophagy under normal conditions 76. However, cells prioritize aerobic glycolysis over OXPHOS during immune activation, which leads to a surge in mtROS levels. This increase can induce mitochondrial damage and the subsequent mtDNA release, which then amplifies inflammatory responses 77.

#### 3.4.1. cGAS-STING

The cGAS-STING (cyclic GMP-AMP synthase-stimulator of interferon genes) is a cytoplasmic DNA-sensing pathway that triggered by type I IFN production 78. Upon binding to double-stranded DNA (dsDNA), cGAS utilizes the formation of cyclic guanosine monophosphate-adenosine monophosphate (cGAMP) using ATP and GTP, which subsequently binds and activates STING 79. Dysregulated cGAS-STING signaling links to a spectrum of inflammatory diseases 80. Therapeutic intervention targeting the cGAS-STING pathway, such as the inhibition of its substrate or catalytic sites, could potentially ameliorate autoimmune disorders 81. STING antagonists can act by occupying its cyclic dinucleotide binding site 82 or by binding covalently to cysteine residue 91 to prevent STING palmitoylation, an essential posttranslational modification for its activity 83.

#### 3.4.2. AIM2 Inflammasome

The AIM2 (absent in melanoma 2) inflammasome functions as a sensor for cytosolic double-stranded DNA that activates inflammatory caspases, engaging the adaptor protein ASC and procaspase-1 to facilitate its assembly. This complex initiates the cleavage and subsequent translocation of gasdermin D to cell membrane 84. The activation of the AIM2 inflammasome is intricately linked to the metabolic reprogramming of immune cells. For example, in septic mice induced by LPS, PKM2-driven glycolysis leads to the phosphorylation of eukaryotic translation initiation factor 2-α kinase 2, which mediates the activation of NLRP3 and AIM2 inflammasome 19. Heightened mtROS levels prompts the assembly of the AIM2 inflammasome, thus the cleavage of procaspase-1 and subsequent cleavage of Parkin, a negative regulator of mitophagy, thereby impeding mtROS clearance and enhancing mitochondrial damage 85. Additionally, AIM2 inflammasome is suppressed in LPS-primed macrophages when the synthesis of 25-hydroxycholesterol is upregulated through cholesterol biosynthesis 86.

The role of the AIM2 inflammasome is context-dependent, varying with cell type and disease. In systemic lupus erythematosus, AIM2 expression is markedly upregulated in leukocytes and macrophages, though not in kidney tissue 87. Conversely, activation of the AIM2 inflammasome aggravates atherosclerosis in individuals with clonal hematopoiesis 88. In contrast, Akt interacts with AIM2 to inhibit the Akt/mTOR/Myc, thus promotes lipid oxidation in mitochondria. This enhances the stability of T_reg_ cells in response to inflammatory stimuli, thereby limiting the development of autoimmunity in experimental autoimmune encephalomyelitis 89.

## 4. Potential Metabolic Regulators

For numerous immunologists and pharmacologists, the most urgent inquiry pertains to the potential for introducing a regulatory layer to oversee cellular metabolic programming, with the objective of controlling innate immune responses. The regulation of cellular metabolic programming has the potential to be a highly efficacious therapeutic intervention, as well as to provide crucial insights into the fundamental relationship between metabolic alterations and the signaling control of innate immunity.

### 4.1. Glycolysis Inhibitors

Small molecules can modulate glycolysis at various enzymatic steps. The compound 2-deoxyglucose (**2-DG**), a structural analog of glucose, competitively inhibits phosphoglucose isomerase, thereby curbing the formation of glucose-6-phosphate, a critical early intermediate in glycolysis 90. The use of 2-DG and the fatty acid synthase inhibitor **C75** has been shown to forestall the activation of DCs by disrupting the glycolysis-driven de novo synthesis of fatty acids 91. Therapeutically, **2-DG** administration attenuates oxidative stress and the systemic inflammatory response in murine models of acute lung injury and septic shock-induced kidney injury 92. Furthermore, a recent Phase II clinical trial has reported that **2-DG**, administered at a dose of 90 mg/kg/day in conjunction with the standard of care, provides additional benefit to patients with COVID-19, compared to the standard treatment alone 93.

Compound 3-(3-pyridinyl)-1-(4-pyridinyl)-2-propen-1-one, a potent inhibitor of PFKFB3, mitigates endothelial inflammation in LPS-induced acute lung injury mice 94. The cell-permeable itaconate derivative 4-octyl itaconate covalently modifies the Cys22 residue on GAPDH to inhibit its glycolytic activity, thus resulting in the amelioration of the inflammatory response within macrophages 95.

Capsaicin interacts directly with Cys424 on PKM2, thereby inhibiting the enzyme's facilitation of the Warburg effect. Treatment with capsaicin, at a dosage of 1 mg/kg, mitigates systemic inflammation and multiple organ dysfunction in a murine model of septic shock induced by LPS 96. The modulation of PKM2-mediated glycolytic metabolism through agents such as iminostilbene or shikonin associates with reduced inflammatory response in macrophages during myocardial ischemia-reperfusion injury 97, 98, and in T_h_17 cells in the context of non-alcoholic fatty liver disease 99. In collagen-induced arthritis mice, Panax notoginseng saponins obstructs STAT3 phosphorylation by preventing nuclear translocation of PKM2, which in turn decreases differentiation of T_h_17 cells 100. Conversely, enhancing PKM2 metabolic function with the allosteric activator TEPP-46 restricts T_h_17 cells maturation, thereby potentially reducing autoimmunity in models of experimental autoimmune encephalomyelitis and multiple sclerosis 20. DASA-58, another well-characterized PKM2 activator 18, impedes glycolysis and the inflammatory response in macrophages triggered by LPS and follistatin-like protein 101. Additionally, a series of coxylanolactone derivatives have been synthesized, among which the compound **D5** is identified as a PKM2 activator. **D5** inhibits T_h_17 cell differentiation, restoring the T_h_17/T_reg_ cell balance and ameliorating symptoms of colitis in mice models induced by sodium glucan sulfate and 2,4,6-tritrobenzene sulfonic acid 102.

### 4.2. NADPH Oxidase Inhibitors

NADPH oxidase is pivotal in catalyzing the reduction of oxygen to superoxide anion, a reaction essential for the oxidative bursts that are a key component of the immune defense system 24. Consequently, the development of NADPH oxidase inhibitors has become an area of intense research focus.

Apocynin, first isolated from the root of *Apocynum cannabinum* in 1908 and subsequently from *Picrorhiz kurroa* in 1971 , was later identified as a selective inhibitor of NADPH oxidase 103. Apocynin alleviates corneal injury and inflammatory response in corneal neovascularization by its ROS scavenging activity 104. Interestingly, apocynin also diminished neutrophil survival by modulating ERK1/2 phosphorylation induced by granulocyte-macrophage colony-stimulating factor (GM-CSF), independent of its inhibitory activity on NADPH oxidase 105. The small molecule LDC7559 and its derivative NA-11 target PFKL, and selectively attenuate NOX-2-dependent oxidative bursts, effectively moderating excessive inflammation in human neutrophils 28.

The NOX1/4 inhibitor GKT137831, also known as setanaxib, potentiates immune activity in CD8^+^ T cells, enhancing their infiltration into cancer-associated fibroblasts and potentially reversing resistance to programmed cell death protein 1 (PD-1)/PD-ligand 1 immunotherapy 106. GKT137831 is currently undergoing Phase IIb/III clinical trials for primary biliary cholangitis and hepatitis steatosis, as well as a Phase II clinical trial for squamous cell carcinoma of the head and neck (Clinical trial No. NCT05014672, NCT05323656).

The NOX5 specific inhibitor ML090 significantly reduces edema and cerebral induced cerebral ischemic injury when administered as a pretreatment, suggesting its potential as a preventative strategy in combination with thrombolytic drugs 107.

### 4.3. ROS Scavengers

The strategic removal of surplus mtROS relies on the development of specialized chemical scavengers. MitoQ, a ubiquinone-derived compound conjugated with a triphenylphosphonium cation, is a lipophilic cation engineered to cross biological membranes and accumulate in the mitochondrial inner membrane, leveraging the mitochondrial membrane potential 108. Clinical studies have demonstrated the efficacy of MitoQ in a range of conditions, including Parkinson's disease 109, neuroinflammation 110, mtDNA damage associated with high-intensity exercise 111, and the enhancement of vascular function in healthy older adults 112.

Tiron, or sodium 4,5-dihydroxybenzene-1,3-disulfonate, represents another mitochondria-targeted antioxidant. It has shown promise in improving airway inflammation in a chronic asthma model in mice, displaying effectiveness comparable to the clinically prescribed corticosteroid, dexamethasone 113. Moreover, Tiron inhibits the activation of the NLRP3 inflammasome in endothelin-1-induced models of erectile dysfunction 114, and offers superior protection against oxidative damage from hydroperoxide and UV radiation in the 315-400 nm range in human skin fibroblasts when compared to MitoQ 115.

Another notable compound is mito2HOBA ((4-(4-aminomethyl)-3-hydroxyphenoxy)butyl)-triphenylphosphonium), a mitochondria-targeted scavenger synthesized by conjugating 2-hydroxybenzylamine with the lipophilic cation triphenylphosphonium. Mito2HOBA significantly diminishes systemic inflammation in LPS-induced septic shock in mice 116.

Augmentation with key NAD^+^ precursors, such as nicotinamide riboside (NR) and nicotinamide mononucleotide (NMN), may activate enzymes critical for NAD biosynthesis. Deficits in NMN and NAD^+^ correlate with metabolic impairments and the enhanced presence of CD38 in immune cells, a phenomenon often observed with aging 117. Long-term supplementation with NMN and NR is linked to a reduction in age-related inflammation and oxidative stress in murine models 117. Clinical trials reveal that NMN can substantially improve insulin sensitivity and signaling in prediabetic women following a daily intake of 250 mg for a duration as brief as ten weeks 118.

However, it is imperative to consider treatment duration, dosing regimens, and potential long-term adverse effects. Current human studies typically use doses of up to 500 mg in the above mentioned studies, which are significantly lower than the doses used in mouse models, where approximately 300 mg per kilogram is common - equivalent to approximately 22.5 g for a 75 kg individual. This striking difference underscores the need for cautious dose extrapolation between species. Experts agree that further research using high-throughput methods is essential to elucidate the effects of NAD^+^ and its precursors on the epigenome, transcriptome, proteome, and metabolome. In addition, long-term administration of NMN or nicotinamide riboside and its effects on healthy individuals warrant rigorous investigation to ensure safety and efficacy.

### 4.4. cGAS Inhibitors

The inhibition of cGAS focuses on attenuating its catalytic function. PF-06928215, the inaugural cGAS inhibitor, was discovered via a fluorescence polarization assay, exhibits high affinity (k*_D_*=200 nM) and potency 81. Enhanced derivatives, including compounds **18**, **S2**, and **S3**, target the catalytic domain of cGAS and demonstrate superior inhibition, as confirmed through a pyrophosphate (PP_i)_-coupled assay and computational screening 119.

Another class of cGAS inhibitors emerged from a screen for compounds that hinder synthesis of cGAMP. RU.521, notable for its potency, binds to Arg364 and Tyr421 of cGAS, engaging the phthalide ring's aldehyde group and forming hydrogen bonds with Gly290 and Lys350 of murine cGAS. Ru.521 uniquely attenuates dsDNA-stimulated type I IFN expression in BMDMs isolated from mice with Aicardi-Goutieres syndrome 120.

Lama *et al.* introduced the small molecules **G108** and **G150** as human cGAS inhibitors, leveraging an ATP-dependent, luminescence-based high-throughput screen. These compounds target the cGAS active site, selectively reducing dsDNA-induced IFN response in human THP-1 cells and primary macrophages 121. Similarly, **Cu-32**, **Cu-76**, and related molecules disrupt the dimer interface of human cGAS, specifically targeting cytosolic DNA-triggered, but not RNA-induced, IFN response 122.

Beyond these targeted molecules, certain approved drugs also exhibit inhibition to cGAS. Antimalarial agents, such as hydroxychloroquine sulfate, chloroquine, and quinine, along with the aminoacridine derivative **X6**, diminish IFN-β production by blocking cGAS-dsDNA interactions 123, 124. Suramin, an established therapy for parasitic diseases, also inhibits cGAS activity without impacting TLR1/TLR2 or TLR4 pathways 125, 126. Currently, suramin is undergoing Phase II trials for acute kidney injury (clinical trial No. NCT04496596), autism, and several types of cancer 127, 128. Additionally, aspirin acetylates cGAS at Lys384, Lys394, or Lys414, and its administration at 50 mg/kg mitigates autoimmunity in models of Aicardi-Goutieres syndrome and in patient-derived peripheral blood mononuclear cells 129.

### 4.5. STING Agonists

Targeting the palmitoylation of STING offers therapeutic promise 130. Nitrofuran derivatives **C-170**, **C-171**, **C-176**, **C-178**, and the indoles derivative **H-151** irreversibly inhibit multimeric STING complexes assembly at the Golgi, curtailing downstream signaling 83. **C-176** specifically mitigates STING-associated inflammatory osteolysis 131. Building on this, Liu et al. subsequently developed **SP23**, a STING-targeting proteolysis-targeting, from **C-170**, effectively dampening inflammation in a murine model of cisplatin-induced acute kidney injury 132. Nitro-fatty acids (NO_2_-FAs), endogenously lipid, alkylate STING at Cys88, Cys91, and His16, impeding its palmitoylation 133.

Electrophilic acrylamide, **BPK-21** and **BPK-25**, binds to Cys91 residue of STING, precluding its signaling activation in human primary T cells. Notably, **BPK-25** also inhibits cGAMP-induced STING activation in peripheral blood mononuclear cells 134. Tetrahydroisoquinolone derivative **18** interacts with Thr263 of STING, fostering an inactive conformation and obstructing cGAMP-mediated cytokine production 135. Astin C, a cyclic peptide, competitively occupies cyclic dinucleotide binding sites, thwarting cGAS-STING signalosome assembly in inflammatory responses in Trex1^-/-^ mice 82. Further, compound **13**, a butenolide heterodimer-based inhibitor, selectively inhibits the cGAS-STING pathway, reducing IFN-β and viral dsDNA-induced gene expression in THP-1 cells 136. These findings indicate that the blockade of the downstream signal pathway is a more efficacious approach to controlling the metabolic changes-induced inflammatory response.

### 4.6. AIM2 Inflammasome Inhibitors

The activation of the AIM2 inflammasome consistently occurs concomitantly with the activation of other inflammasomes during viral infections, thus AIM2 inhibitors are anticipated to be used in combination with other agents that modulate the immune response. Several compounds have been identified to repress the AIM2 inflammasome-mediated immune response, including CRID3 137, shikonin 138, compound **J114** 139, and the bisphenol compound obovatol 140. Thus far, no AIM2-specific inhibitors have been reported.

### 4.7. Metabolic Checkpoint Inhibitors

Metabolic checkpoints play a pivotal role in the regulation of the innate immune response, thereby ensuring the optimal functioning of immune cells such as macrophages and dendritic cells. Principal metabolic regulators include AMP-activated protein kinase (AMPK) and mTOR, which are capable of sensing cellular energy status and nutrient availability 141. AMPK activation facilitates the catabolic pathways that generate ATP, thereby supporting the survival and function of innate immune cells under conditions of low energy. Conversely, mTOR stimulates anabolic processes, promoting cell growth, proliferation, and effector functions in response to nutrient abundance. The nuclear factor of activated T-cells (NFAT), which is activated by calcineurin, plays a role in the innate immune response by regulating the production of pro-inflammatory cytokines 142. Immunosuppressive agents such as rapamycin inhibit the activity of mTOR, which in turn reduces the inflammatory activity of innate immune cells 143. This can be beneficial in conditions such as sepsis, organ transplantation, and chronic inflammation. The modulation of these metabolic checkpoints by pharmacological agents demonstrates the intricate interplay between metabolism and innate immune regulation, thereby providing potential therapeutic strategies for inflammatory and immune-mediated diseases.

### 4.8. Combination Therapies

Recent studies highlight the promise of combining metabolic modulators with immune-targeted therapies to counteract pathological metabolic adaptations in immune cells. In cancer immunotherapy, glycolysis inhibitors (e.g., 2-DG) or monocarboxylate transporter 1 (MCT1) inhibitors (AR-C155858, MCT1i) synergize with anti-PD-1 antibodies to alleviate lactate-driven immunosuppression and reverse T cell exhaustion, enhancing antitumor responses 144, 145. Similarly, MCT1 inhibitors like AZD3965 improve chimeric antigen receptor T-cell efficacy in B-cell malignancies by mitigating metabolic competition in the tumor microenvironment 146. In psoriasis, the combination of IL-17 antibodies with soraphen A, an acetyl-CoA carboxylase (ACC) inhibitor, targets the metabolic reprogramming of γδT17 cells. These cells shift toward aerobic glycolysis and ATP-citrate synthase-dependent fatty acid synthesis under inflammatory conditions. The blocking of ACC by soraphen A has been shown to disrupt fatty acid synthesis, deplete lipid stores, and suppress IL-17A production in γδT17 cells. This, in turn, has been demonstrated to potentiate the therapeutic effect of IL-17 inhibition 147. These examples underscore the importance of multi-pathway engagement to overcome metabolic plasticity in immune cells.

## 5. Future Prospective and Challenges

In future research, several key areas are likely to significantly impact the therapeutic approaches targeting cellular metabolic programming. Firstly, a comprehensive analysis of the intricate interconnections between pathways such as glycolysis, lipid metabolism, and the pentose phosphate pathway will provide a more nuanced understanding of metabolic programming, which in turn will inform the development of more practical therapeutic strategies. Computational modeling and artificial intelligence are emerging as powerful tools to decipher complex metabolic networks and predict therapeutic outcomes, enabling researchers to identify critical nodes for intervention. Secondly, the relationship between mitochondrial function and metabolic reprogramming needs to be thoroughly explored, which is of particular importance in the context of modulating innate immune responses and facilitate the development of more efficacious treatments.

Another significant challenge in the field is the need to account for interspecies differences in immune regulation, particularly between murine models and human physiology, to enhance the translational potential of preclinical findings. Challenges include metabolic redundancy, off-target effects (e.g., NOX inhibitors affecting non-immune cells), and interspecies variability limiting translational potential. Personalized approaches may be needed to account for patient-specific metabolic profiles. The advent of personalized medicine approaches, meticulously tailored to individual metabolic and immune profiles, plays a pivotal role in optimizing therapeutic efficacy and minimizing adverse effects. This assertion is particularly pronounced in the context of immune-mediated diseases, given their inherent heterogeneity. A paucity of human data exists regarding DUOX isoforms in IBD, as well as the role of TCA metabolites in chronic inflammation. Moreover, the majority of combination therapies are still in the preclinical stage, emphasizing the necessity for clinical validation.

In conclusion, the advent of new technologies and applications provides researchers with powerful tools for advancing metabolic reprogramming research. The integration of immunology, metabolism, bioinformatics, and clinical medicine can facilitate a comprehensive understanding of metabolic reprogramming. Techniques such as single-cell sequencing, mass spectrometry, and CRISPR gene editing are of pivotal importance for the uncovering of detailed molecular mechanisms and the identification of potential therapeutic targets. Continued research, coupled with innovative technologies and interdisciplinary collaboration, demonstrates considerable potential for translating metabolic reprogramming into groundbreaking therapies for immune-related diseases.

## Figures and Tables

**Figure 2:**
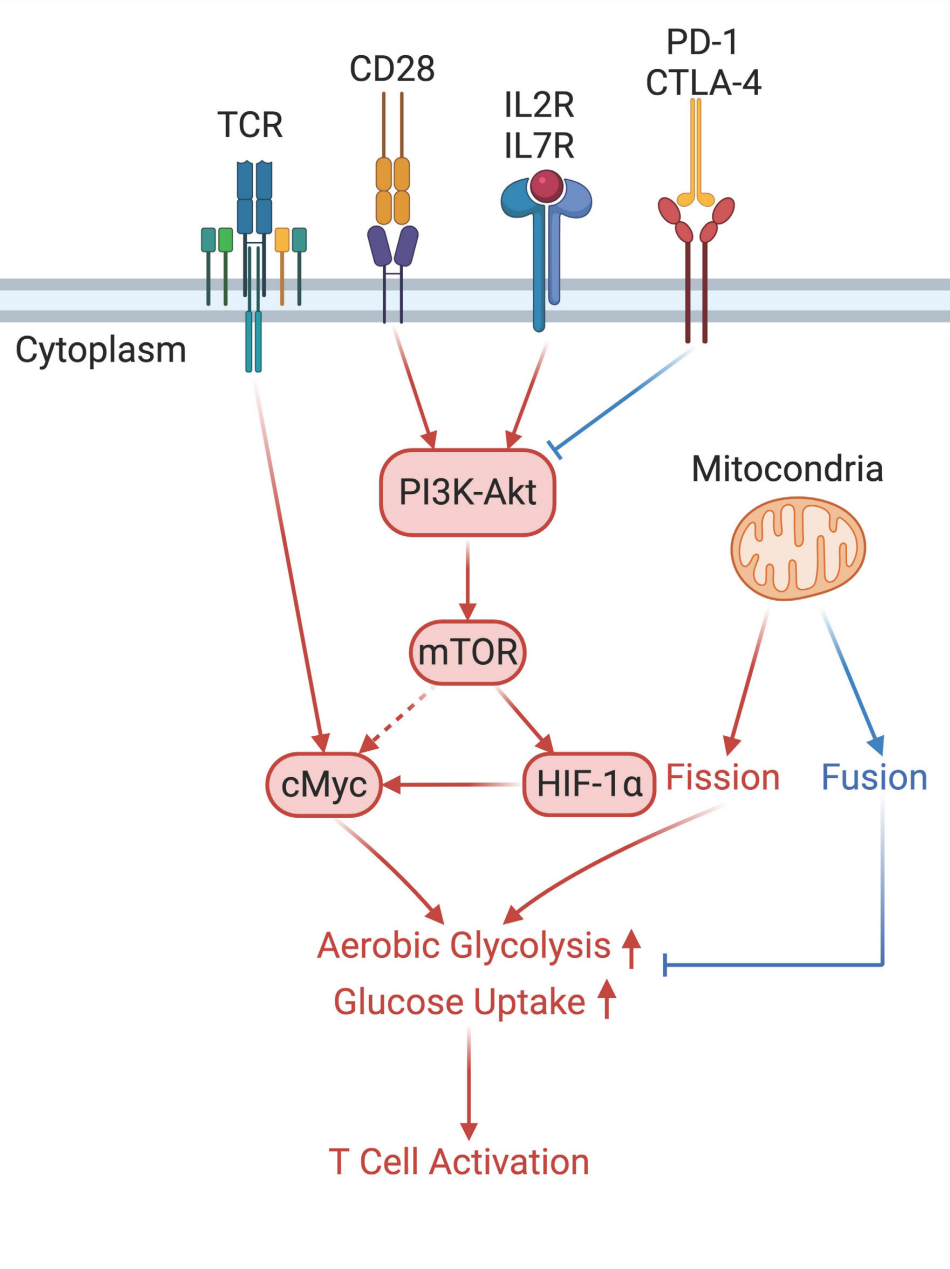
In the T cell activation cascade, the T cell receptor (TCR), along with co-stimulatory and co-receptor molecules like CD28, IL2R, and IL7R, orchestrates activation via the PI3K-Akt-cMyc axis. In contrast, programmed cell death protein 1 (PD-1) and cytotoxic T-lymphocyte-associated protein 4 (CTLA-4) serve as inhibitory checkpoints, dampening T cell activation by modulating the PI3K-Akt pathway. The diagrammatic representation employs red arrows to denote the pathways promoting T cell activation, while blue arrows indicate the pathways conferring inhibitory signals that antagonize activation.

**Figure 3:**
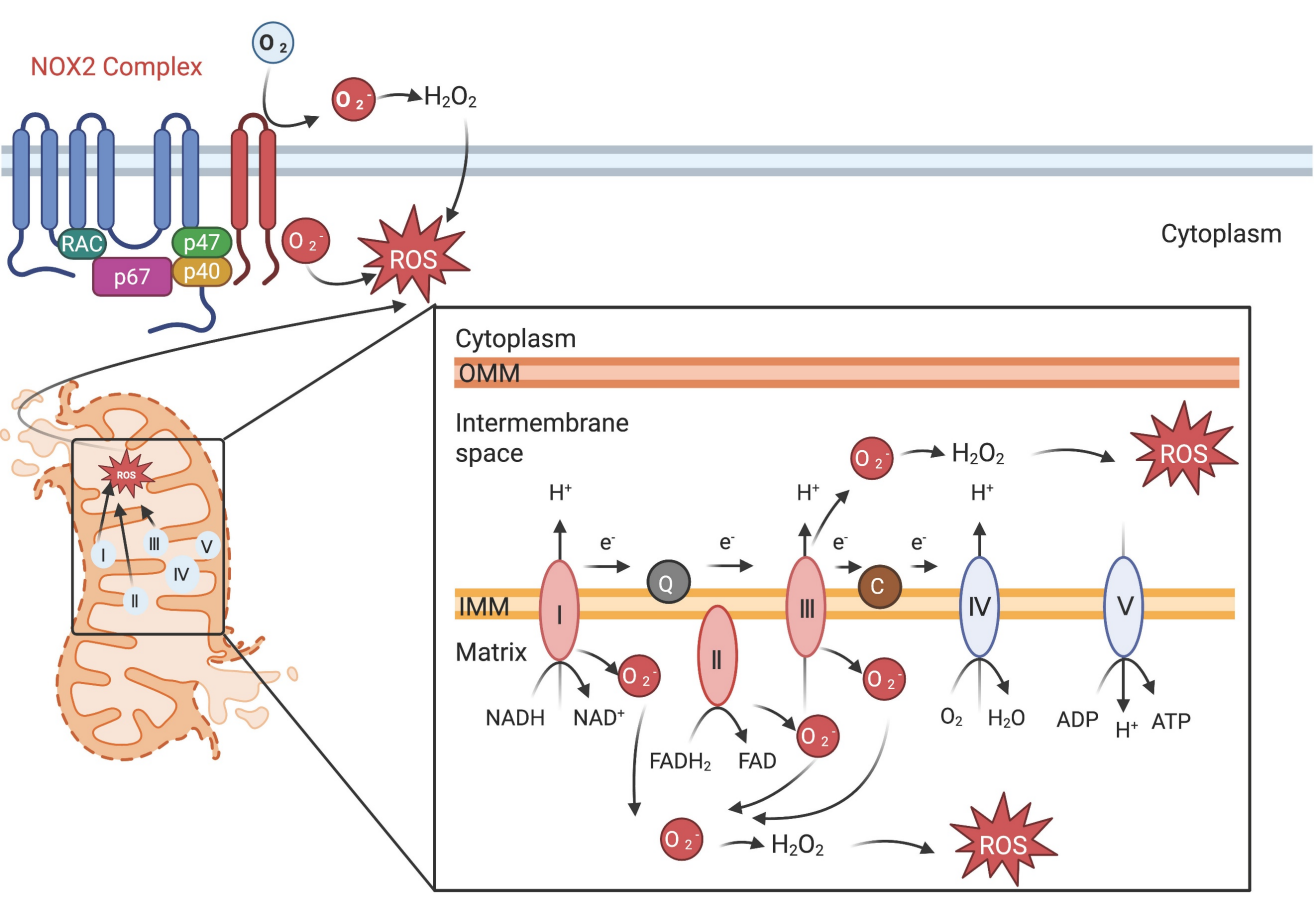
Mitochondria and NOX2 complexes are responsible for the generation and release of ROS into the cytoplasm. Mitochondrial Complexes I to III together with NOX2 complex generates O2-, a precursor to a multitude of ROS.

**Figure 4:**
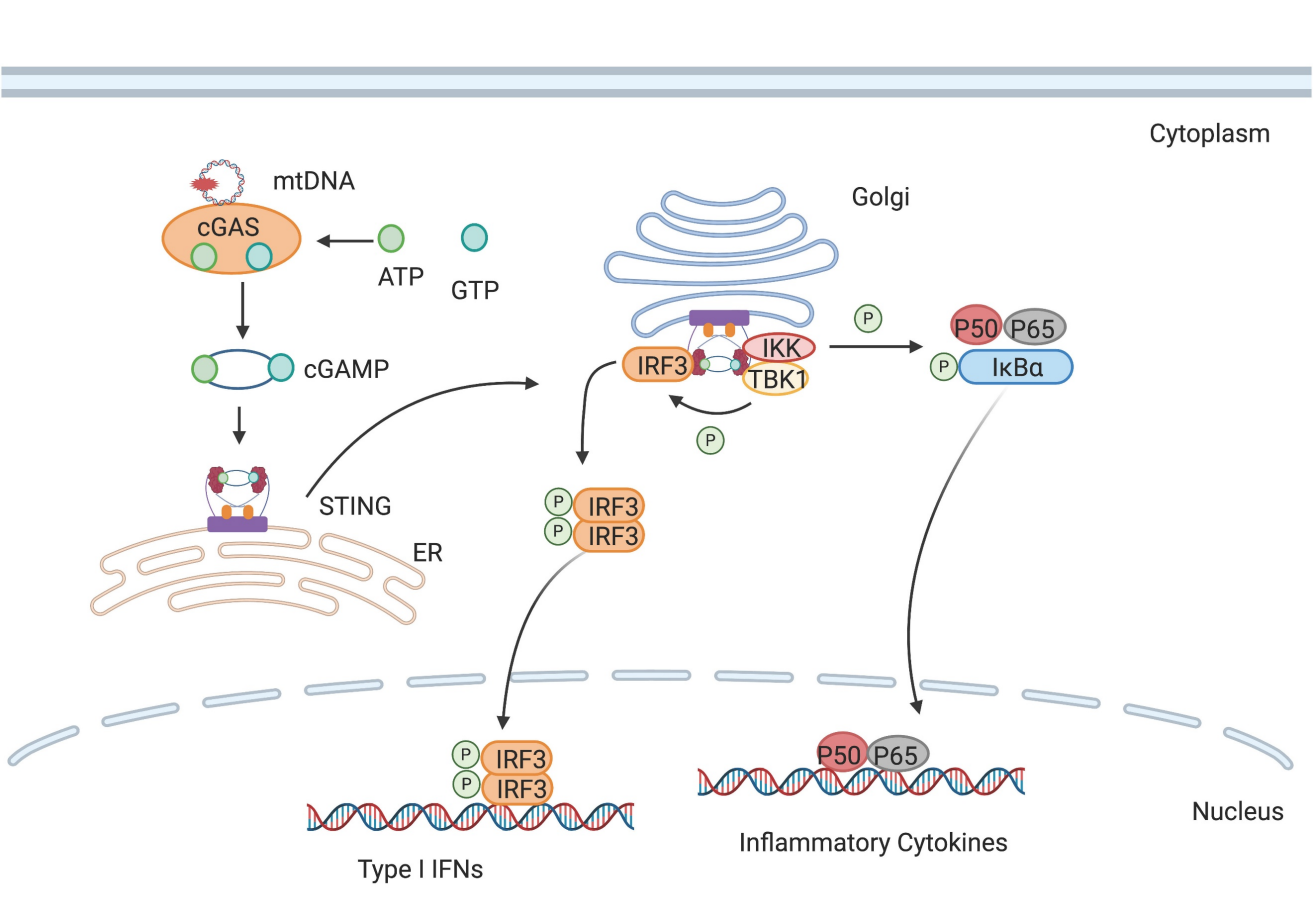
cGAS is activated by mtDNA, thereby catalyzing the formation of cGAMP. cGAMP binds to STING and promotes its transfer from the endoplasmic reticulum to the Golgi apparatus, subsequently activating downstream inflammatory pathways.

**Figure 5:**
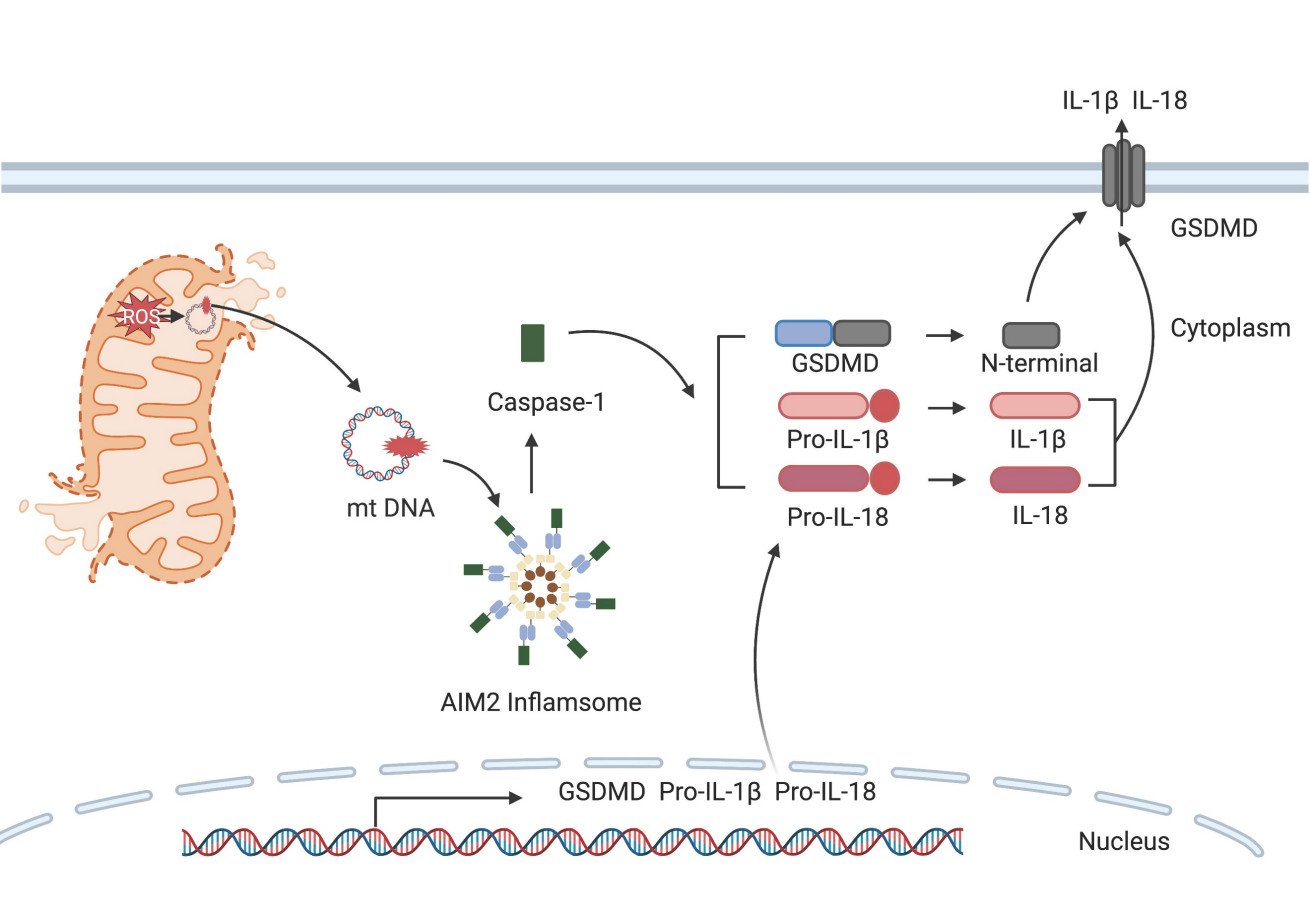
The release of mtDNA from damaged mitochondria into the cytoplasm stimulates the activation of AIM2 inflammasome. Caspase-1 subsequently cleaves gasdermin D to generate N-terminal fragments that assemble into membrane pores, while also maturing pro-IL-1β and pro-IL-18 for release through these channels.

**Table 1:** Inhibitors that control metabolic signaling.

Inhibitor	Chemical Structure	Mode of Regulation	Disease Types	References
**Glycolysis inhibitors**				
2-DG	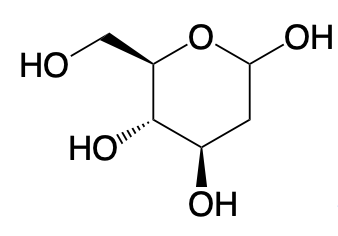	Competitively inhibits the production of glucose-6-phosphate	Acute lung and kidney injury in mice; COVID-19 and herpes simplex virus infected patients	91-93
3-(3-pyridinyl)-1-(4-pyridinyl)-2-propen-1-one	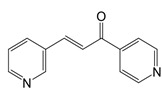	Binds to PFKFB3 in a dose-dependent manner	Acute lung injury mice model	88
4-Octyl itaconate	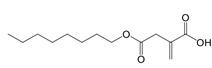	Binds to Cys22 of GAPDH	Endotoxaemia mice model	95
Capsaicin	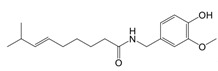	Binds to Cys424 of PKM2	Septic shock mice model; neuropathic pain and amyotrophic lateral sclerosis patients	96
iminostilbene or shikonin	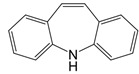 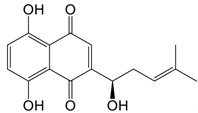	Binds to PKM2 in a dose-dependent manner	Myocardial ischemia-reperfusion injury and non-alcoholic fatty liver disease mice models	97, 99
TEPP-46	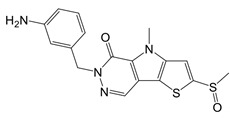	Promotes PKM2 tetramer formation	Encephalomyelitis and multiple sclerosis mice models	20
DASA-58	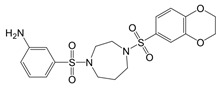	Promotes PKM2 tetramer formation	Hepatic fibrosis mice model	18, 101
D5	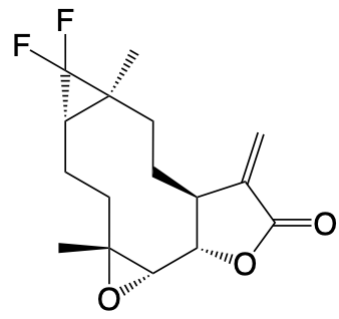	Promotes PKM2 tetramer formation	Ulcerative colitis mice model	102
**NADPH oxidase inhibitors**				
Apocynin	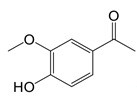	Blocks p47phox membrane translocation	Corneal alkali burn mice model; sodium-induced declines in cutaneous microvascular function bronchial asthma patients	104, 105, 148
LDC7559 /NA-11	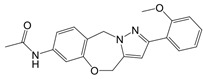 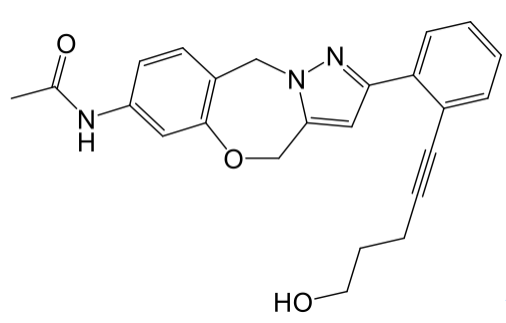	Binds to the AMP/ADP allosteric activation site	Neutrophil-induced bronchial epithelial damage mice model	28
GKT137831 (Setanaxib)	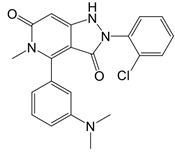	Direct inhibitor of NOX1 and NOX4	Type 2 diabetes and primary biliary cholangitis patients	Clinical trial No. NCT02010242, NCT03226067
ML090	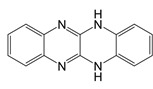	NOX5 inhibitor	Stroke patients	107
**ROS Scavenger**				
MitoQ	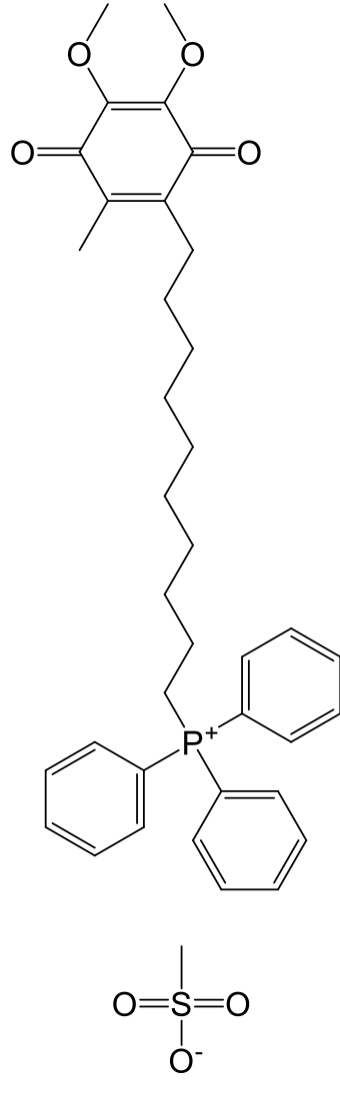	A mitochondria-targeted antioxidant	Tissue hypoxia induced by neurological deficits in mice; improve vascular function Parkinson's disease exercise-induced mitochondrial DNA damage in patients	108-112
Tiron (sodium 4,5-dihydroxybenzene-1,3-disulfonate)	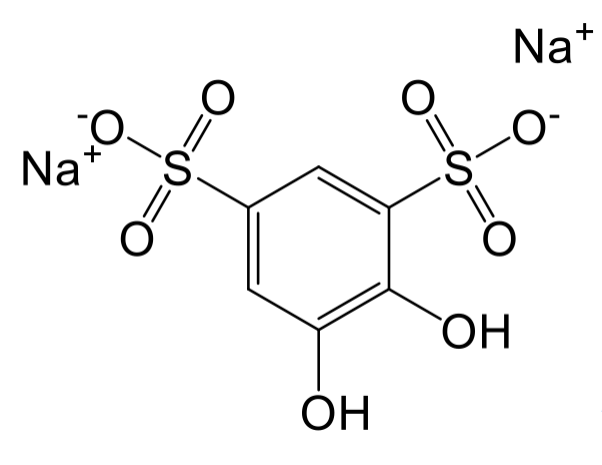	A mitochondria-targeted antioxidant	Airway remodeling and erectile dysfunction in mice	113-115
mito2HOBA (4-(4-aminomethyl)-3-hydroxyphenoxy)butyl)-triphenylphosphonium)	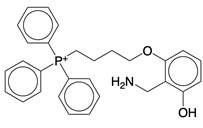	A mitochondria-targeted isolevuglandins scavenger	N/A	116
nicotinamide mononucleotide (NMN) and nicotinamide riboside (NR)	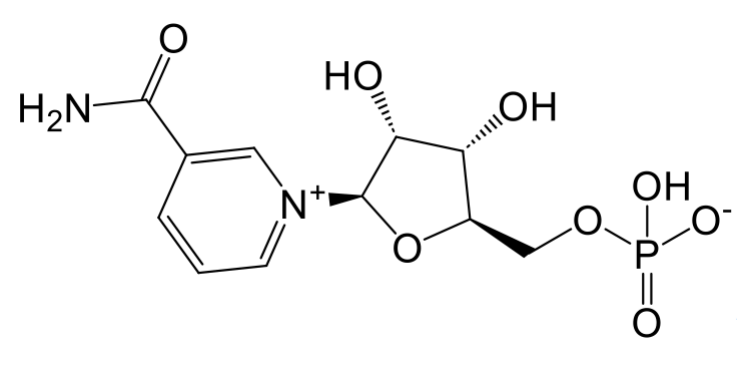 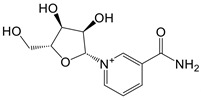	Activate enzymes control biosynthesis of NAD	Aged mice; and prediabetes patients	117, 118
**Itaconate derivative**				
4-Octyl itaconate	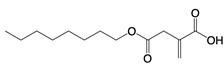	Protects against lethality and systemic inflammation induced by LPS	Septic shock mice model	69
**cGAS inhibitors**				
PF-06928215	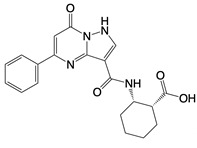	Binds to the catalytic domain of cGAS	Aged mice and high fat diet-induced cardiac anomalies in mice	81
Compounds 18, S2, and S3	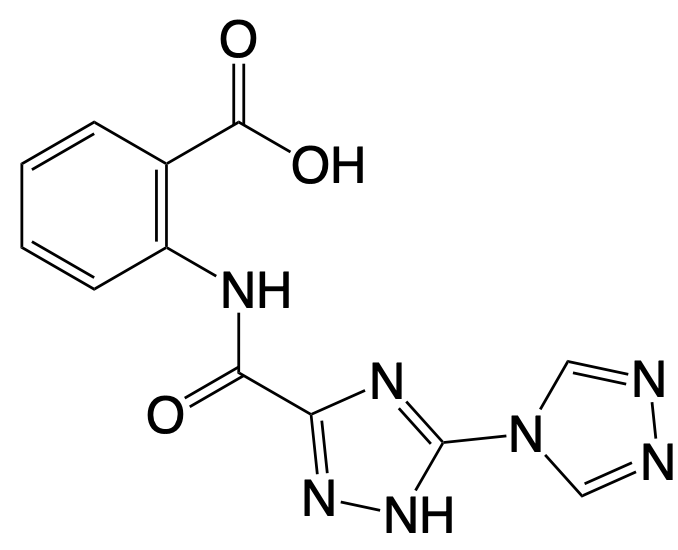 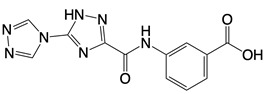 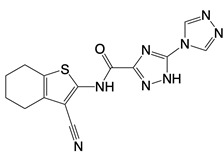	Binds to the catalytic domain of cGAS	N/A	119
RU.521	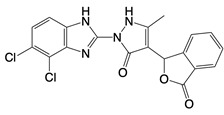	Binds to residues Arg364 and Tyr421 of cGAS	Subarachnoid hemorrhage-induced brain injury, cerebral venous sinus thrombosis, acute liver injury, rheumatoid arthritis, and postoperative cognitive dysfunction models in mice	120
G108 and G150	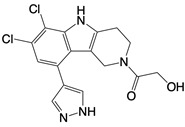 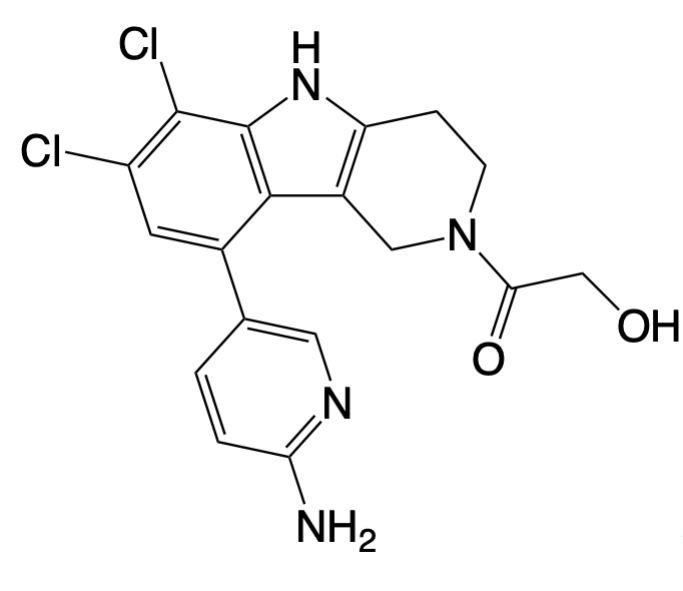	Binds to the active site of cGAS	N/A	121
Cu-32, Cu-76, and their analogs	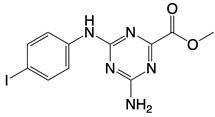 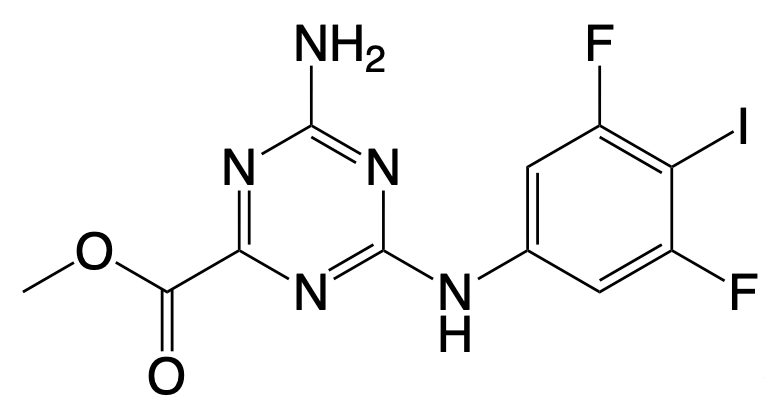	Binds to the dimer interface of human cGAS	N/A	122
hydroxychloroquine sulfate, chloroquine, and quinine	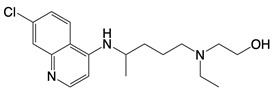 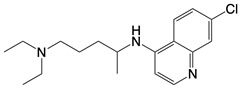 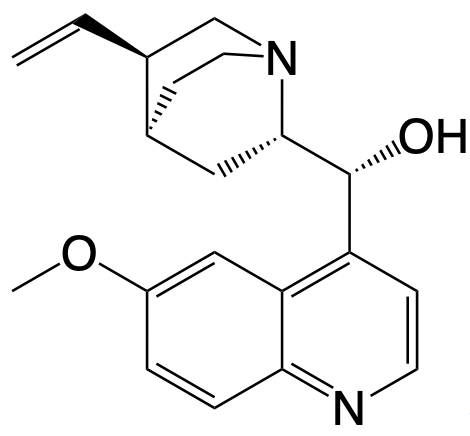	Inhibits cGAS upon dsDNA stimulation of	Antineoplastic effects in mice; rheumatoid arthritis, systemic lupus rythematosus, and SARS-CoV-2 infection in patients	123
X6	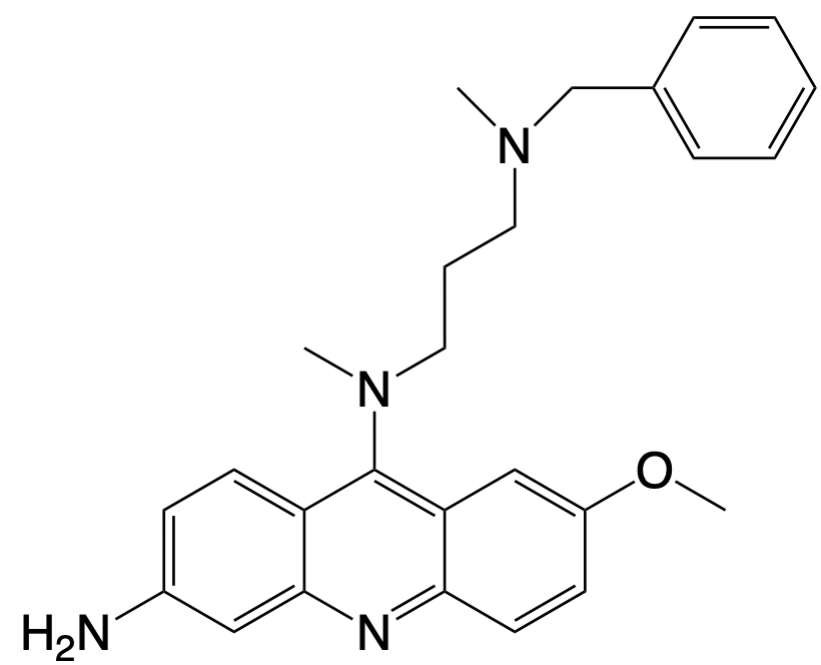	Blocks interaction of cGAS to dsDNA	N/A	124
Suramin	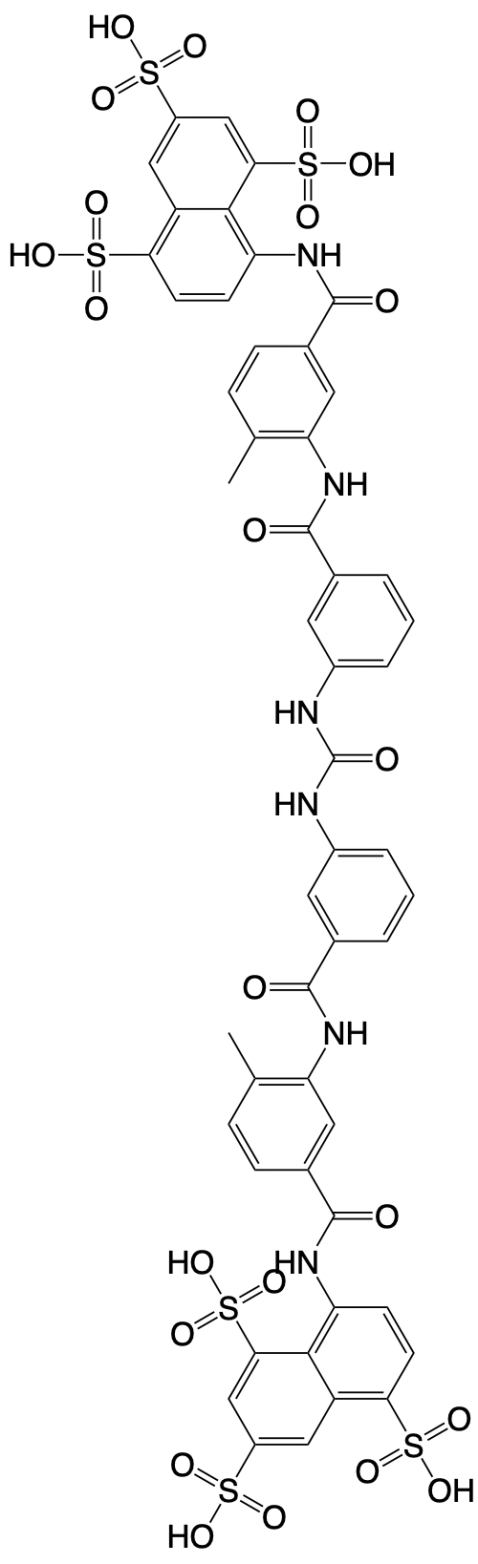	Inhibits the enzymatic activity of cGAS	Osteoarthritis, chikungunya virus infection, and diabetic nephropathy in mice	127, 128, Clinical trial No. NCT04496596
Aspirin	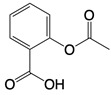	Binds to residues Lys384, Lys394, or Lys414 of cGAS	Atherosclerotic cardiovascular disease, diabetes, and periodontitis in mice; patients with atherosclerotic cardiovascular disease	129
**STING inhibitor**				
Nitrofuran derivative C-170, C-171, C-176, C-178, and the indoles derivative H-151	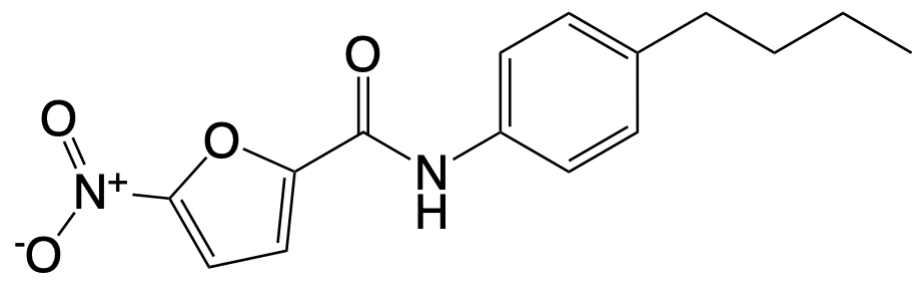 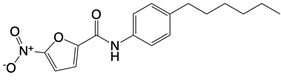 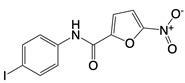 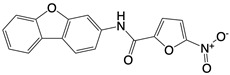 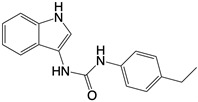	Disrupt assembly of the multimeric STING complexes	N/A	83, 131
SP23	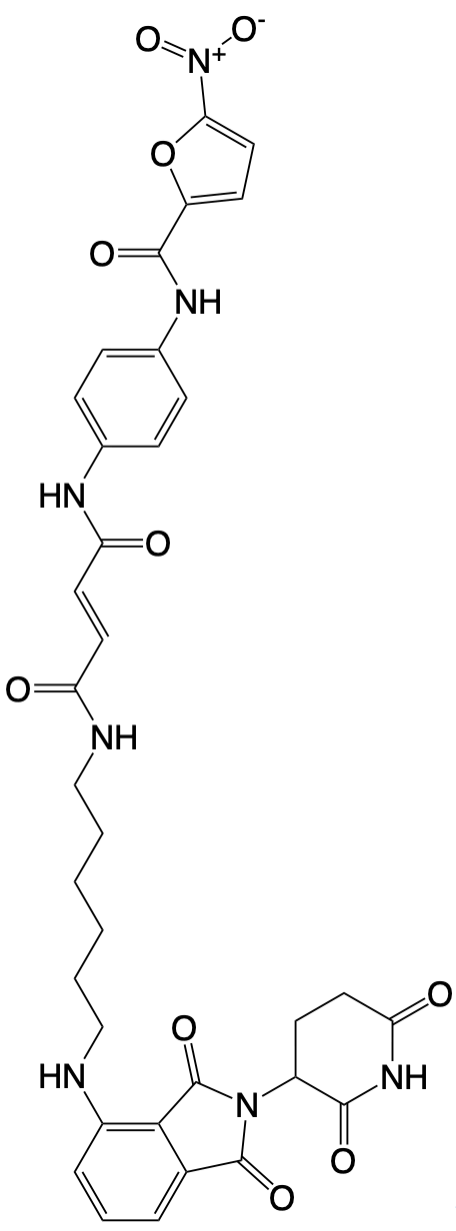	Blocks assembly of the multimeric STING complexes	Mice renal cell carcinoma model	132
Nitro-fatty acids (NO_2_-FAs)	Not disclosed	Binds to residues Cys88, Cys91, and His16 of STING	Mice myocardial fibrosis model	133
BPK-21 and BPK-25	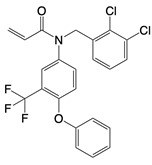 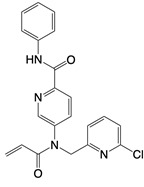	Binds to residue Cys 91 of STING	N/A	134
Compound 18	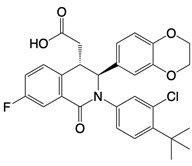	Binds to residues Thr263 and Thr267 of STING	N/A	135
Astin C	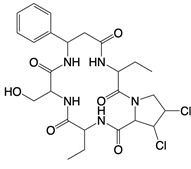	Binds to the cyclic dinucleotide sites of STING	Colitis and cardiac anomalies in mice	82
Compound 13	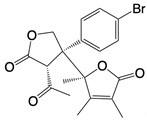	A pathway-specific antagonists of cyclic GMP-AMP synthase	N/A	136
**AIM2 inhibitors**				
CRID3	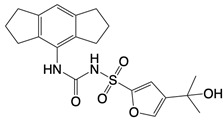	Inhibits formation of ASC complexes	Spinal cord injury in mice	137
Shikonin	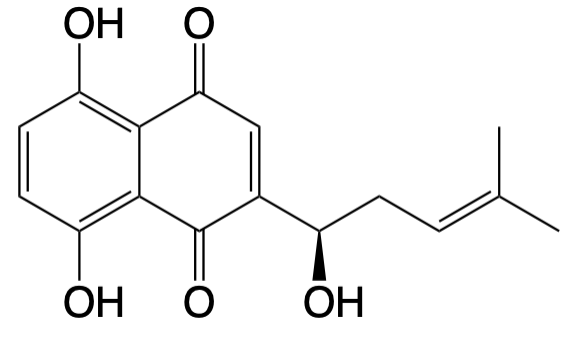	Dampens formation of ASC specks and directly inhibit caspase-1 enzymatic activity	Acute liver injury, ovarian cancer, skin diseases, wound healing, and lung cancer in mice	138
J114	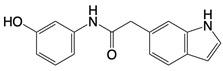	Blocks interaction between AIM2 and ASC and inhibit ASC oligomerization	Mice keratitis model	139
Obovatol	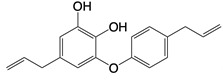	Inhibits formation of ASC pyroptosome	Mice Alzheimer's disease, colorectal cancer, bone disorders, and hepatocellular carcinoma models	140
**mTOR inhibitor**				
Rapamycin	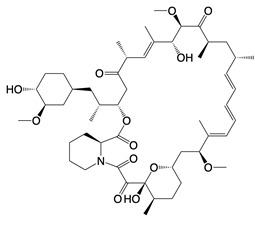	Inhibits PI3K-Akt signaling, AMPK and mTOR activity	Glaucoma, lung injury, and aging in mice; tuberous sclerosis complex-associated tumors in patients	141, 149

## Abbreviations

2-DG: 2-deoxyglucose
ACC: acetyl-CoA carboxylase
AIM2: absent in melanoma 2
Akt: serine/threonine kinase 1
AMPK: AMP-activated protein kinase
ASC: PYD and CARD domain containing
BMDM: bone marrow-derived macrophage
CD28: cluster of differentiation 28
cGAMP: cyclic guanosine monophosphate-adenosine monophosphate
cGAS: cyclic GMP-AMP synthase
DCs: dendritic cells
dsDNA: double-stranded DNA
DUOX: dual oxidase
ETC: electron transport chain
F2,6BP: fructose-2,6-bisphosphate
F6P: fructose 6-phosphate
FADH_2_: flavin adenine dinucleotide
FUNDC1: fun14 domain-containing protein 1
G3P: glycerol-3-phosphate
G6P: glucose 6-phosphate
GAPDH: glyceraldehyde-3-phosphate dehydrogenase
GLUT: glucose transporters
GM-CSF: granulocyte-macrophage colony stimulating factor
GSDMD: gasdermin d
GTP: guanosine-5'-triphosphate
HIF-1α: hypoxia-inducible factor-1α
HK: hexokinase
IBD: inflammatory bowel disease
IFN: interferon
IKK: inhibitor of nuclear factor κ B kinase
IL: interleukin
IMM: inner mitochondrial membrane
iNOS: inducible nitric oxide synthase
IRAK: IL-1R-associated kinases
IRF: interferon regulatory factor
IκB: inhibitor of nuclear factor κ B
LC3: microtubule-associated protein 1A/1B-light chain 3
LDHA: lactate dehydrogenase A
LPS: lipopolysaccharide
MCT1: monocarboxylate transporter 1
MitoQ: mitoquinol mesylate
mtDNA: mitochondrial DNA
mTOR: mammalian target of rapamycin
mtROS: mitochondrial reactive oxygen species
NAD: nicotinamide adenine dinucleotide
NADH: nicotinamide adenine dinucleotide+hydrogen
NADPH: triphosphopyridine nucleotide hydrogen
NF-κB: nuclear factor κ B
NLRC4: NOD-like receptor family card domain containing 4
NLRP3: NOD-like receptor family pyrin domain containing 3
NMN: nicotinamide mononucleotide
NOX: NADPH oxidase
NQO: quinone oxidoreductases
NR: nicotinamide riboside
OMM: outer mitochondrial membrane
OXPHOS: oxidative phosphorylation
PD-1: programmed cell death protein 1
PEP: 2-phosphoenolpyruvate
PFK1: 6-phosphofructokinase-1
PFK2: 6-phosphofructo-2-kinase
PFKFB3: fructose-2,6-biphosphatase 3
PFKL: phosphofructokinase-1 liver type
PI3K: phosphoinositide 3-kinase
PKM2: pyruvate kinase isozymes M2
RAC: Ras-related C3 botulinum toxin substrate
RET: reverse electron transport chain
ROS: reactive oxygen species
SARS: severe acute respiratory syndrome
siRNA: small interfering RNA
STING: stimulator of interferon genes
TBK: tank-binding kinase
TCA cycle: tricarboxylic acid cycle
TCA: tricarboxylic acid
TCR: T cell receptor
T_h_ cell: T helper cell
TLR: Toll-like receptor
TNF-α: tumor necrosis factor-α
T_reg_ cell: regulatory T cell
